# Joint meetings: Exchange of ideas and knowledge advancement

**DOI:** 10.4103/1817-1737.30352

**Published:** 2007

**Authors:** Hamdan Al-Jahdali

**Affiliations:** *Chairman, Scientific committee, The 5^th^ Annual Scientific Conference of The Saudi Thoracic Society and The 26^th^ Regional Conference of IUALTD*

The recent Joint Conference of the Saudi Thoracic Society (STS) and pan-Arab Thoracic Society meeting held in Jeddah, Saudi Arabia, in September 2006 was the first combined educational effort to simultaneously bring together two major societies with similar vision and goals; this has set the stage for more collaboration in the future with other national, regional and international professional societies in the same field of interest.

The fifth STS Annual Scientific Meeting scheduled to be held in Riyadh, Saudi Arabia, from March 20 to 22, 2007, will be another milestone for the STS for many reasons, the most significant being that it will be the first joint meeting between the STS and the International Union Against Tuberculosis and Lung Disease (IUATLD). These joint meetings offer tremendous opportunity for the exchange of ideas; advancement of knowledge; and, equally important, renewal of friendships among professionals interested in the field of pulmonary disease, thoracic surgery, intensive care and respiratory care, who work so far away from one another.

This upcoming conference will coincide with the International Tuberculosis (TB) day, and this is reflected in the scientific program, where there will be more than one session on multiple days devoted to issues related to TB.

Distinguished speakers will present the updated information on TB provided by the IUATLD and World Health Organization (WHO) and will share their experience and knowledge with the audience from all over the region. Tuberculosis is a major global problem. Overall, one-third of the world's population is currently infected with the tuberculosis bacillus. Worldwide, 8 million new cases of tuberculosis are estimated to occur annually, and there are an estimated 2 million deaths a year. In the eastern Mediterranean region, tuberculosis is an important public health problem. Almost a third of the population is infected with *Mycobacterium tuberculosis*, with an estimated incidence of 122 per 100,000 population and an estimated mortality rate of 28 per 100,000. In 2004, 206,160 new cases were notified.[1]

The resurgence of tuberculosis is one of the most serious global public health challenges of the 21^st^ century. The decline of tuberculosis since the 19^th^ century is far better understood than its resurgence over the last 20 years.[2]

Widespread implementation of the Directly Observed Treatment Short course (DOTS strategy) is the major progress in global tuberculosis control seen in the past decade, but still there are many challenges hindering global tuberculosis control. Human immunodeficiency virus and multi-drug-resistant (MDR) TB and, recently, Extensive Drugs Resistance tuberculosis (XDR) are the major challenges, mainly in poor countries such as African and Far East countries. A recent study from one of the Middle Eastern countries estimates that XDR occurs in approximately 11% of MDR TB.[3] During this meeting, there will be discussions about many aspects of pulmonary disease, but special emphasis will be given to tuberculosis. There will be discussions on Millennium Development Goals, stop-TB strategy, prevention and control of extreme multi-drug-resistant tuberculosis and other topics. Furthermore, there will be many simultaneous workshops on TB, thoracic surgery, sleep medicine, mechanical ventilation and many more subjects.
